# Supplementary material for: Comparative multiomics analysis of cell physiological state after culture in a basket bioreactor
Source: Sci Rep. 2022 Nov 23;12:20161. doi: 10.1038/s41598-022-24687-4 (PMC9686226; doi:10.1038/s41598-022-24687-4)
Supplement: Supplementary file 1 — Supplementary Information 1. [file 41598_2022_24687_MOESM1_ESM.zip › raw data/Transcriptomic raw data/6.Enrichment/2.KEGG/TE1vsTC1/ALL/src/csab04721.html]

csab04721


- csab:103246629

- Down regulated genes

103246629(-1.52295540054731)

- csab:103246502

- Down regulated genes

103246502(-2.12412044998812)

- csab:103239806

- Down regulated genes

103239806(-1.24609138243098)

- csab:103239806

- Down regulated genes

103239806(-1.24609138243098)

- csab:103246502

- Down regulated genes

103246502(-2.12412044998812)

- csab:103246629

- Down regulated genes

103246629(-1.52295540054731)

Close
